# Predicting response to immunotherapy plus chemotherapy in patients with esophageal squamous cell carcinoma using non-invasive Radiomic biomarkers

**DOI:** 10.1186/s12885-021-08899-x

**Published:** 2021-10-30

**Authors:** Ying Zhu, Wang Yao, Bing-Chen Xu, Yi-Yan Lei, Qi-Kun Guo, Li-Zhi Liu, Hao-Jiang Li, Min Xu, Jing Yan, Dan-Dan Chang, Shi-Ting Feng, Zhi-Hua Zhu

**Affiliations:** 1 Department of Thoracic Surgery, Sun Yat-sen University Cancer Center; State Key Laboratory of Oncology in South China; Collaborative Innovation Center for Cancer Medicine, Guangzhou, 510080 Province Guangdong, People’s Republic of China; 2 Department of Radiology, The First Affiliated Hospital of Sun Yat-sen University, Guangzhou, 510080 Province Guangdong, People’s Republic of China; 3 Department of Interventional Oncology, The First Affiliated Hospital of Sun Yat-sen University, Guangzhou, 510080 Province Guangdong, People’s Republic of China; 4 Department of Thoracic Surgery, The First Affiliated Hospital of Sun Yat-sen University, Guangzhou, 510080 Province Guangdong, People’s Republic of China; 5 Department of Radiological Interventional, The First Affiliated Hospital of Sun Yat-sen University, Guangzhou, 510080 Province Guangdong, People’s Republic of China; 6 Department of Medical Imaging Center, Sun Yat-sen University Cancer Center; State Key Laboratory of Oncology in South China, Collaborative Innovation Center for Cancer Medicine, Guangzhou, 510080 Province Guangdong, People’s Republic of China; 7 Scientific Collaboration, CT-MR Division, Canon Medical System (China), Jiuxianqiao North Road, Chaoyang District, 100015, Beijing, People’s Republic of China

**Keywords:** Esophageal cancer, Radiomics, Tomography, X-ray computed, Immunotherapy

## Abstract

**Objectives:**

To develop and validate a radiomics model for evaluating treatment response to immune-checkpoint inhibitor plus chemotherapy (ICI + CT) in patients with advanced esophageal squamous cell carcinoma (ESCC).

**Methods:**

A total of 64 patients with advance ESCC receiving first-line ICI + CT at two centers between January 2019 and June 2020 were enrolled in this study. Both 2D ROIs and 3D ROIs were segmented. ComBat correction was applied to minimize the potential bias on the results due to different scan protocols. A total of 788 features were extracted and radiomics models were built on corrected/uncorrected 2D and 3D features by using 5-fold cross-validation. The performance of the radiomics models was assessed by its discrimination, calibration and clinical usefulness with independent validation.

**Results:**

Five features and support vector machine algorithm were selected to build the 2D uncorrected, 2D corrected, 3D uncorrected and 3D corrected radiomics models. The 2D radiomics models significantly outperformed the 3D radiomics models in both primary and validation cohorts. When ComBat correction was used, the performance of 2D models was better (*p* = 0.0059) in the training cohort, and significantly better (*p* < 0.0001) in the validation cohort. The 2D corrected radiomics model yielded the optimal performance and was used to build the nomogram. The calibration curve of the radiomics model demonstrated good agreement between prediction and observation and the decision curve analysis confirmed the clinical utility.

**Conclusions:**

The easy-to-use 2D corrected radiomics model could facilitate noninvasive preselection of ESCC patients who would benefit from ICI + CT.

**Supplementary Information:**

The online version contains supplementary material available at 10.1186/s12885-021-08899-x.

## Background

Esophageal cancer (EC) is the seventh most common cancer cause of death in male population worldwide [1]. China accounts for more than half of the world’s new cases and EC-related deaths, with more than 90% diagnosed EC being esophageal squamous cell carcinoma (ESCC) [2]. Surgery, chemotherapy and radiotherapy are the cornerstone treatments of EC [3, 4]. However, outcomes are still poor with a 5-year survival rate of 10–15% [5]. The emerging targeted drugs used to treat EC are only targeting HER2 or vascular endothelial growth factor [6–8], and the therapeutic effect of improved traditional treatments with added targeted drugs is still unsatisfactory with a 5-year survival rate of 30–40% for ESCC [9]. Therefore, there is a high clinical need for novel and more effective treatment options for EC patients.

In recent years, the study of KEYNOTE-028 and KEYNOTE-180 first confirmed the efficacy and safety of pablizumab in the treatment of advanced EC [10, 11]. Whereafter, in a larger sample size, KEYNOTE-181 established the position of pablizumab in the treatment of advanced EC [12]. At present, a number of studies have been performed to explore the efficacy and safety of immunotherapy combined with chemotherapy as first-line and post-line treatment of advanced ESCC [13–16]. The comprehensive positive score (CPS), tumor proportion score (TPS) are immunohistochemical markers for evaluating the expression of programmed death receptor ligand 1 (PD-L1) in tumors. However, the precision of these biomarkers was unsatisfying. Therefore, more reliable biomarkers for predicting the efficacy of immunotherapy for EC is in urgent need.

With the rapid development of artificial intelligence (AI) in the field of medical imaging, radiographic characteristics of tumors referred to as ‘radiomics’ have shown success in immunotherapeutic response prediction for different tumor types [17–19]. To the best of our knowledge, there is no evidence yet in EC. In this study, we aimed to evaluate the potential predictive value of CT-derived radiomics in advanced ESCC patients receiving immune-checkpoint inhibitor plus chemotherapy (ICI + CT).

## Methods

### Study design

**Fig. 1:**
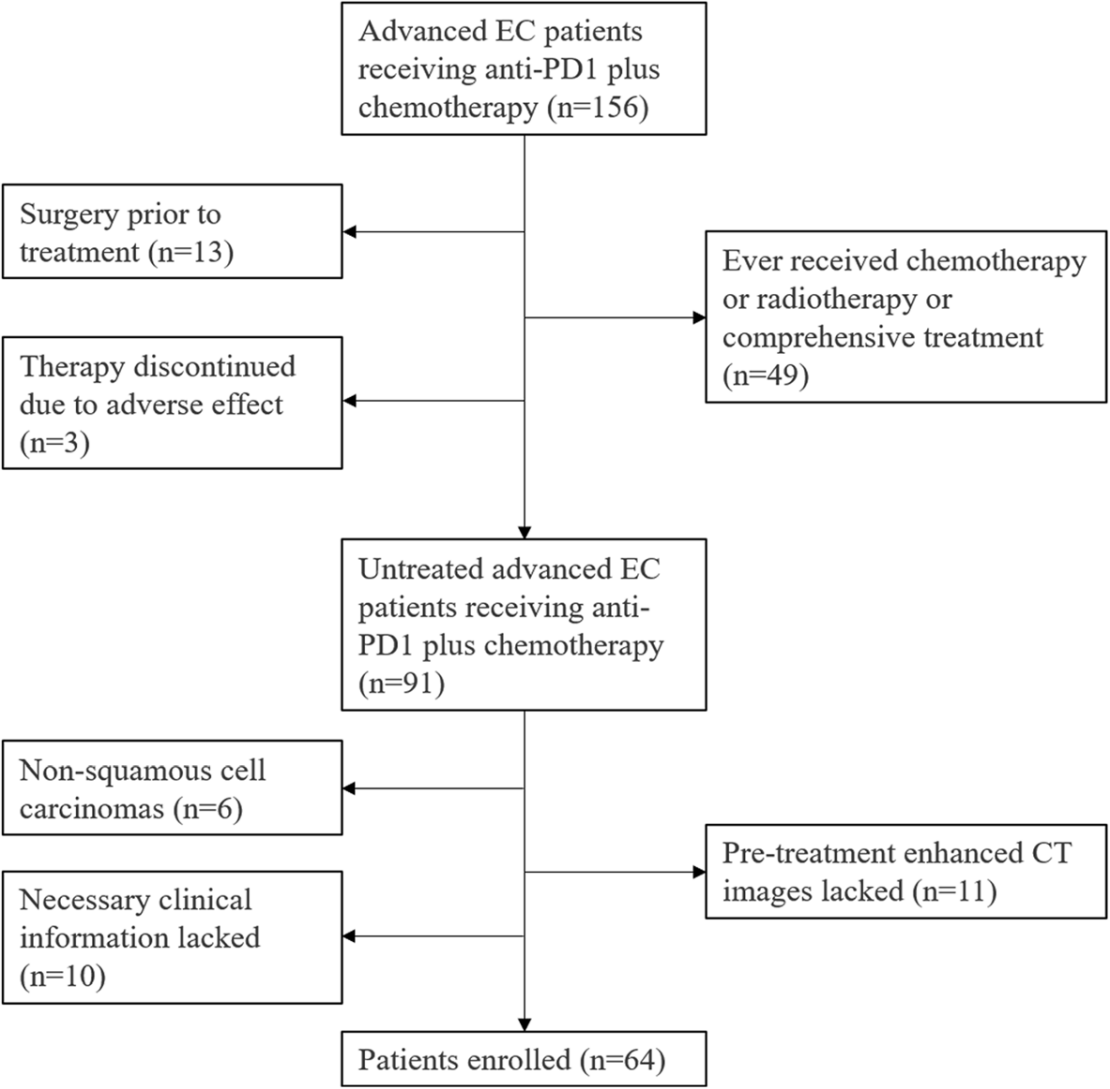
Flowchart of Patient enrollment

A total of 64 patients with advance inoperable ESCC receiving 200 mg every 3 weeks of Sintilimab plus Docetaxel (60 mg/m^2^) and Carboplatin (AUC = 5) at two centers between January 2019 and June 2020 were included in this study approved by the two institutional review boards. All patients in two centers had undergone MDT study before starting treatment. Informed consent was waived. In this study patients were confirmed by biopsy and immunohistochemistry of the original tumor tissue. All the enrolled patients were first-visit and prior to treatment. Patients who had never received cancer related treatments including radiotherapy, chemotherapy, comprehensive treatment and surgery and those who lacked CT imaging data and necessary clinical information before the initial treatment (immunotherapy plus chemotherapy) were excluded from this study. Exclusion criteria also included patients with non-squamous cell carcinoma including adenocarcinoma and signet ring cell carcinoma and those who discontinued treatment due to adverse events. Flow chart of patient enrollment is shown in Fig. 1. For patients’ clinical characteristics, information of age, gender, Body Mass Index (BMI), clinical TNM stage, hemoglobin, blood albumin, leucocyte, C-reactive protein and underlying diseases was acquired from electronic medical record system. BMI was calculated based on height and weight. Clinical TNM stage was confirmed by pre-treatment gastroscopy, CT examination, etc.

### Response kinetics and scan protocol

Contrast enhanced computed tomography (CE-CT) scans were acquired before (baseline) and around six weeks (two cycles) after start of treatment (follow-up). Treatment response was evaluated by assessing the relative change in diameter between baseline and follow-up, using RECIST 1.1 criteria [20]. Patients were divided into responders [complete/partial response disease] and non-responders [stable and progressive disease] according to RECIST. For progressive disease, pseudoprogression was confirmed by follow-up observation.

All preoperative enhanced CT images were obtained with multidetector CT scanners during inspiration. Detailed information of the CT scanners including manufacturer, country of origin, tube voltage, slice thickness and spacing was shown in Supplementary Table 1. Iopromide (300 mg I/m1, Schering Pharmaceutical Ltd) was used as the contrast agent for enhanced scanning protocol, and 80–100 ml was injected at 3–4 ml/s flow rate.

### Lesion segmentation and Radiomics features extraction

All enhanced CT images were manually segmented with an open-source software ITK-SNAP (http://www.itksnap.org/pmwiki/pmwiki.php) for feature extraction. 2D ROI was selected as the slice with maximum axial diameter of the tumor, and 3D ROI was segmented slice by slice on the whole volume of the lesions.

To correct variability from spatial information in three axes (x, y, z) and different CT protocols, all enrolled CT images were resampled to a same isotropic voxel spacing. Considering the distribution of our data, we resampled the 2D ROIs to 1 × 1 mm^2^, and the 3D ROIs to 1 × 1 × 1 mm^3^ to balance between the loss of in-plane information and the interpolation of out-of-plane information. Afterwards, the CT radiomics features, from 2D and 3D ROIs respectively, were extracted with an open-source python platform Pyradiomics (version 2.1.2, https://pyradiomics.readthedocs.io/en/latest/#). Features used in this study included 14 shape-based features, 18 first order statistics features and 68 texture features containing the gray-level co-occurrence matrix (GLCM, 22 features), gray level run length matrix (GLRLM, 16 features), gray level size zone matrix (GLSZM, 16 features) and gray level dependence matrix (GLDM, 14 features). Besides the original images, eight filters were also generated for feature extraction, including wavelet transform filter. All the categories of features other than shape originated from the original and filtered images were calculated. Therefore, in this study, a total of (18 + 68 + 14) + (18 + 68) *8 = 788 features were statistically analyzed.

To control the potential bias caused by various imaging acquisition protocols on the prediction efficacy of the model, ComBat correction method (https://github.com/Jfortin1/ComBatHarmonization) was applied to 2D and 3D ROIs, resulting in four different groups of features for comparison: (1) 2D uncorrected radiomics features; (2) 2D corrected radiomics features; (3) 3D uncorrected radiomics features; (4) 3D corrected radiomics features.

### Feature selection

Feature selection was performed separately for each group of features. Three steps were applied to reduce dimensionality: (1) features with variance larger than 0.8 were included for further analysis; (2) univariate feature selection was done by ANOVA (continuous variable) or chi-square test (discrete variable) to explore the associations between features and treatment response. The features with *p* value>0.05 would be excluded from further analysis; (3) the most significant features were selected by the least absolute shrinkage and selection operator (LASSO) method. Since the total patient number was limited, the nonzero feature coefficients ranking the first five were selected for each group to avoid overfitting.

### Prediction models and workflow

After feature selection, traditional machine learning algorithms, including support vector machine (SVM), k nearest neighbors, random forest, decision tree (DT), logistic regression (LR), were applied to build prediction radiomics models for each feature group. The performance of the models was compared by using 5-fold cross-validation in the validation cohort, with the best model being selected. All the patients were randomly split into 80% for training and the remaining 20% for validation, with 100 iterations. All feature selection and radiomics algorithm selection were based on the data in the training dataset to ensure independence from validation dataset.

**Fig. 2:**
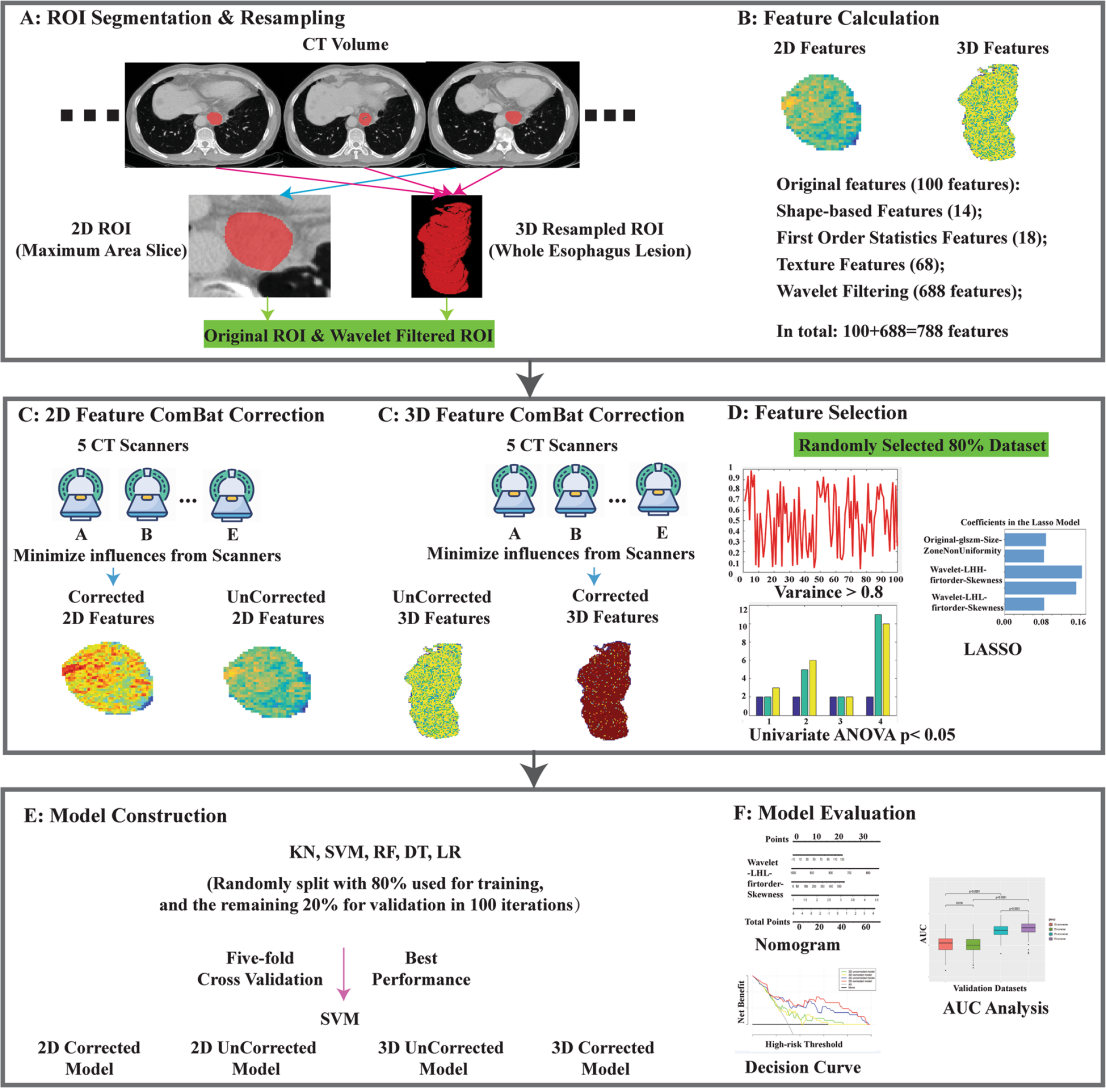
Flowchart of feature selection and radiomics nomogram building. (A) Lesion segmentation and 2D ROI and 3D ROI segmentation; (B) A total of 788 selected features for 2D and 3D ROI respectively; (C) ComBat correction was applied to minimize the potential bias on the results due to different scan protocols of the 5 different CT scanners; (D) Dimension reduction for features selection; (E) Select the optimal algorithm for radiomics model building. The best one was selected by using 5-fold cross-validation in the validation cohort. All the patients were randomly split into 80% for training and the remaining 20% for validation, with 100 iterations; (F) Radiomic nomogram was built on the optimal algorithm. Calibration curves and decision curves were used to evaluate the effectiveness of the radiomics nomogram

Radiomic nomogram was built based on the multivariable logistic analysis of the selected radiomics features in the training group. Calibration curves accompanied by the Hosmer–Lemeshow test were plotted to evaluate the effectiveness of the radiomics nomogram. Decision curve analysis was conducted to determine the clinical usefulness of the radiomics nomogram by quantifying the net benefits at different threshold probabilities in the validation dataset. Flow chart of radiomics nomogram building was illustrated in Fig. 2.

### Statistical analysis

Statistical analyses were performed by using SPSS 22.0 (IBM, USA). Variables were described as frequency (n%). The chi-square test was used to compare patients’ basic information between groups (responders versus non-responders) and *P* < 0.05 was considered statistically significant. All machine learning analyses were performed by using the Python package scikit-learn (0.19.0), and statistical plots were generated by R software (3.6.1, http://www.R-project.org). Area under the Receiver-Operating Characteristic Curves (AUCs) were calculated to evaluate the performance of the algorithms for each model, and the Youden Index was used to generate the optimal threshold to convert probabilities into binarized labels. Statistical metrics, including accuracy, sensitivity, specificity, NPV (Negative Predictive Value), PPV (Positive Predictive Value) and AUC were also calculated to evaluate the performance of the ultimate selected algorithm in the training cohort and the validation cohort for the different radiomics models. Wilcoxon rank test with Bonferroni correction was applied for multiple comparisons, and *p* < 0.0125 was considered statistically significant.

## Results

### Basic Clinicopathological characteristics

A total of 64 patients were included in our study, including 32 (50%) responders and 32 (50%) non-responders. Patients’ clinicopathological characteristics were given in Table 1. No significant difference was observed in underlying diseases between non-responders and responders both in the training and validation cohorts, with *P* value>0.05 respectively.

**Table 1:** Clinicopathological characteristics of advanced ESCC patients treated with ICI + CT

Characteristics	Total	Non-Responders	Responders	***P***
	(***n*** = 64)	(***n*** = 32)	(n = 32)	
Age, year				0.206 †
<60	37 (57.8%)	16 (50%)	21 (65.6%)	
≥ 60	27 (42.2%)	16 (50%)	11 (34.4%)	
Gender, n (%)				1.000 †
Female	10 (15.6%)	5 (15.6%)	5 (15.6%)	
Male	54 (84.4%)	27 (84.4%)	27 (84.4%)	
BMI, n (%)				1.000 †
<18.5	4 (6.3%)	2 (6.3%)	2 (6.3%)	
≥ 18.5 and <24	46 (71.9%)	23 (71.9%)	23 (71.9%)	
≥ 24	14 (21.9%)	7 (21.9%)	7 (21.9%)	
T stage, n (%)				0.633 §
T1	3 (4.7%)	1 (3.1%)	2 (6.3%)	
T2	11 (17.2%)	5 (15.6%)	6 (18.8%)	
T3	31 (48.4%)	18 (56.3%)	13 (40.6%)	
T4	19 (29.7%)	8 (25%)	11 (34.4%)	
N stage, n (%)				0.585 †
N1	18 (28.1%)	10 (31.3%)	8 (25%)	
N2	24 (37.5%)	10 (31.3%)	14 (43.8%)	
N3	22 (34.4%)	12 (37.5%)	10 (31.3%)	
Metastasis, n (%)	19 (29.7%)	13 (40.6%)	6 (18.8%)	0.055 †
Decreased hemoglobin, n (%)	6 (9.4%)	4 (12.5%)	2 (6.3%)	0.668 §
Normal albumin, n (%)	64 (100%)	32 (100%)	32 (100%)	NA
Increased leucocyte, n (%)	10 (15.6%)	5 (15.6%)	5 (15.6%)	1.000 †
C-reactive protein≥10 mg/L, n (%)	31 (48.4%)	13 (40.6%)	18 (56.3%)	0.211 †
Underlying diseases, n (%)	21 (32.8%)	10 (31.3%)	11 (34.4%)	0.790 †

Abbreviations: ICI + CT-Immune-Checkpoint Inhibitor plus Chemotherapy, BMI-Body Mass Index, NA-Not Applicable. † − Pearson chi-square test, §-Fisher’s Exact Test

### Features and optimal Radiomics algorithm selection

For the four different radiomics models including 2D uncorrected, 2D corrected, 3D uncorrected and 3D corrected models, feature selections were performed respectively, and the selected features and their descriptions were shown in Table 2. Algorithms of SVM, KN, RF, and LR were applied to build radiomics models for 2D and 3D ROIs by using selected features from the training cohort, and their performances were compared. The results showed that relatively higher AUC (0.804, 95% CI: 0.800–0.822) could be obtained by using SVM algorithm for the training dataset (Supplementary Table 2). Finally, SVM with the best performance was selected for further evaluation of the performance of radiomics models.

**Table 2:** Selected features of the four different models

Models	Selected radiomic features	Description
3D uncorrected	Wavelet_HHL_glcm_ClusterShade	Skewness and uniformity measurement
	Wavelet_LLH_glszm_SizeZoneNonUniformity	Variability of size zone volumes
	Wavelet_LHH_firstorder_Maximum	Maximum gray level intensity of the ROI
	Wavelet_HHL_firstorder_Skewness	Asymmetry of the mean value
	Wavelet_LLL_gldm_GrayLevelNonUniformity	Variability of gray-level intensity values
3D corrected	Wavelet_LHH_firstorder_Maximum	Maximum gray level intensity of the ROI
	Wavelet_HHL_glcm_ClusterShade	Skewness and uniformity measurement
	Wavelet_LLH_gldm_GrayLevelNonUniformity	Variability of gray-level intensity values
	Wavelet_LLH_glszm_SizeZoneNonUniformity	Variability of size zone volumes
	Wavelet_HLH_glszm_SizeZoneNonUniformity	Variability of size zone volumes
2D uncorrected	Wavelet_HLL_glszm_LargeAreaGrayLevelEmphasis	Proportion in the image of the joint distribution of larger size zones with lower gray-level values
	Wavelet_LHH_firstorder_Skewness	Asymmetry of the mean value
	Original_glszm_SizeZoneNonUniformity	Variability of size zone volumes
	Wavelet_LHL_gldm_DependenceVariance	Variance in dependence size in the image
	Wavelet_LHL_firstorder_Skewness	Asymmetry of the mean value
2D corrected	Wavelet_HLL_firstorder_Skewness	Asymmetry of the mean value
	Wavelet_LHL_firstorder_Maximum	Maximum gray level intensity of the ROI
	Wavelet_LLH_glcm_ClusterProminence	skewness and asymmetry of the GLCM
	Wavelet_LHL_gldm_DependenceVariance	Variance in dependence size in the image
	Original_glszm_SizeZoneNonUniformity	Variability of size zone volumes

### Radiomics models performance based on SVM algorithm

To evaluate the performance of our models in classifying patients according to their treatment response, we used the SVM algorithm. Good performance of the four different radiomics models using SVM algorithm was observed for the probability of responders (Table 3). The results showed that the 2D corrected radiomics model yielded the optimal performance with an AUC of 0.818 [95% CI, 0.797–0.829], an accuracy of 80.4% (95% CI, 79.3–81.5%), a sensitivity of 72.7% (95% CI, 70.6–74.2%), a specificity of 88.6% (95% CI, 85.5–90.0%), a NPV of 79.5% (95% CI, 78.4–80.3%), a PPV of 91.7% (95% CI, 89.6–92.5%) in the training cohort, and an AUC of 0.787 [95% CI, 0.752–0.806], an accuracy of 79.6% (95% CI, 77.0–80.6%), a sensitivity of 71.4% (95% CI, 67.3–76.7%), a specificity of 87.2% (95% CI, 84.1–90.1%), a NPV of 75.3% (95% CI, 72.1–78.6%), a PPV of 84.8% (95% CI, 81.3–87.5%) in the validation cohort.

**Table 3:** Performance evaluation of the radiomic models using SVM algorithm in the training and validation cohort

	Models	Accuracy	Sensitivity	Specificity	NPV	PPV	AUC
Training cohort	3D uncorrected	0.701	0.590	0.814	0.720	0.734	0.626
		(0.690–0.718)	(0.570–0.622)	(0.796–0.831)	(0.700–0.735)	(0.702–0.754)	(0.602–0.637)
	3D corrected	0.690	0.581	0.814	0.705	0.752	0.628
		(0.680–0.702)	(0.556–0.607)	(0.792–0.834)	(0.694–0.721)	(0.720–0.776)	(0.583–0.611)
	2D uncorrected	0.801	0.693	0.900	0.779	0.915	0.776
		(0.800–0.821)	(0.681–0.715)	(0.886–0.932)	(0.771–0.799)	(0.910–0.932)	(0.772–0.791)
	2D corrected	0.804	0.727	0.886	0.795	0.917	0.818
		(0.793–0.815)	(0.706–0.742)	(0.855–0.900)	(0.784–0.803)	(0.896–0.925)	(0.797–0.829)
Validation cohort	3D uncorrected	0.640	0.431	0.864	0.602	0.750	0.531
		(0.632–0.666)	(0.36–0.49)	(0.813–0.900)	(0.575–0.631)	(0.694–0.811)	(0.502–0.560)
	3D corrected	0.640	0.432	0.861	0.601	0.750	0.514
		(0.631–0.660)	(0.363–0.491)	(0.800–0.911)	(0.570–0.632)	(0.691–0.811)	(0.480–0.544)
	2D uncorrected	0.790	0.709	0.860	0.710	0.852	0.729
		(0.770–0.801)	(0.681–0.756)	(0.830–0.891)	(0.564–1.000)	(0.830–0.881)	(0.711–0.760)
	2D corrected	0.796	0.714	0.872	0.753	0.848	0.787
		(0.770–0.806)	(0.673–0.767)	(0.841–0.901)	(0.721–0.786)	(0.813–0.875)	(0.752–0.806)

Abbreviations: SVM-Support Vector Machine, AUC-Area under the Receiver-Operating Characteristic Curve, NPV-Negative Predictive Value, PPV-Positive Predictive Value

**Fig. 3:**
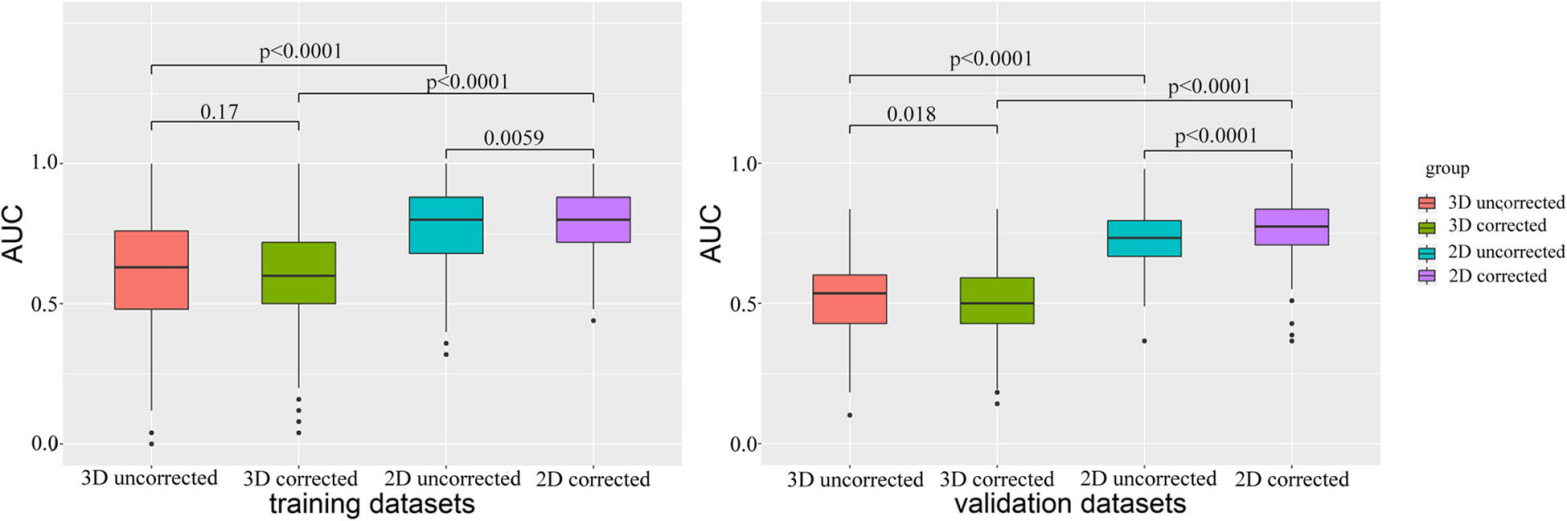
Comparison of AUCs between the four different radiomics models based on SVM algorithm in the training and validation cohort

The performance of the four different radiomics models was compared by AUCs as shown in Fig.3. The 2D models outperformed the 3D models (2D uncorrected vs. 3D uncorrected, *p* < 0.0001; 2D corrected vs. 3D corrected, p < 0.0001) in the training cohort, which was confirmed in the validation cohort (2D uncorrected vs. 3D uncorrected, p < 0.0001; 2D corrected vs. 3D corrected, p < 0.0001). When the ComBat correction was used, the performance of 2D models was better (*p* = 0.0059) in the training cohort, and significantly better (p < 0.0001) in the validation cohort. There was no improvement for 3D models when integrated with the ComBat correction (training cohort, *p* = 0.17; validation cohort, *p* = 0.018).

### Development, performance and validation of individualized Radiomics nomogram

**Fig. 4:**
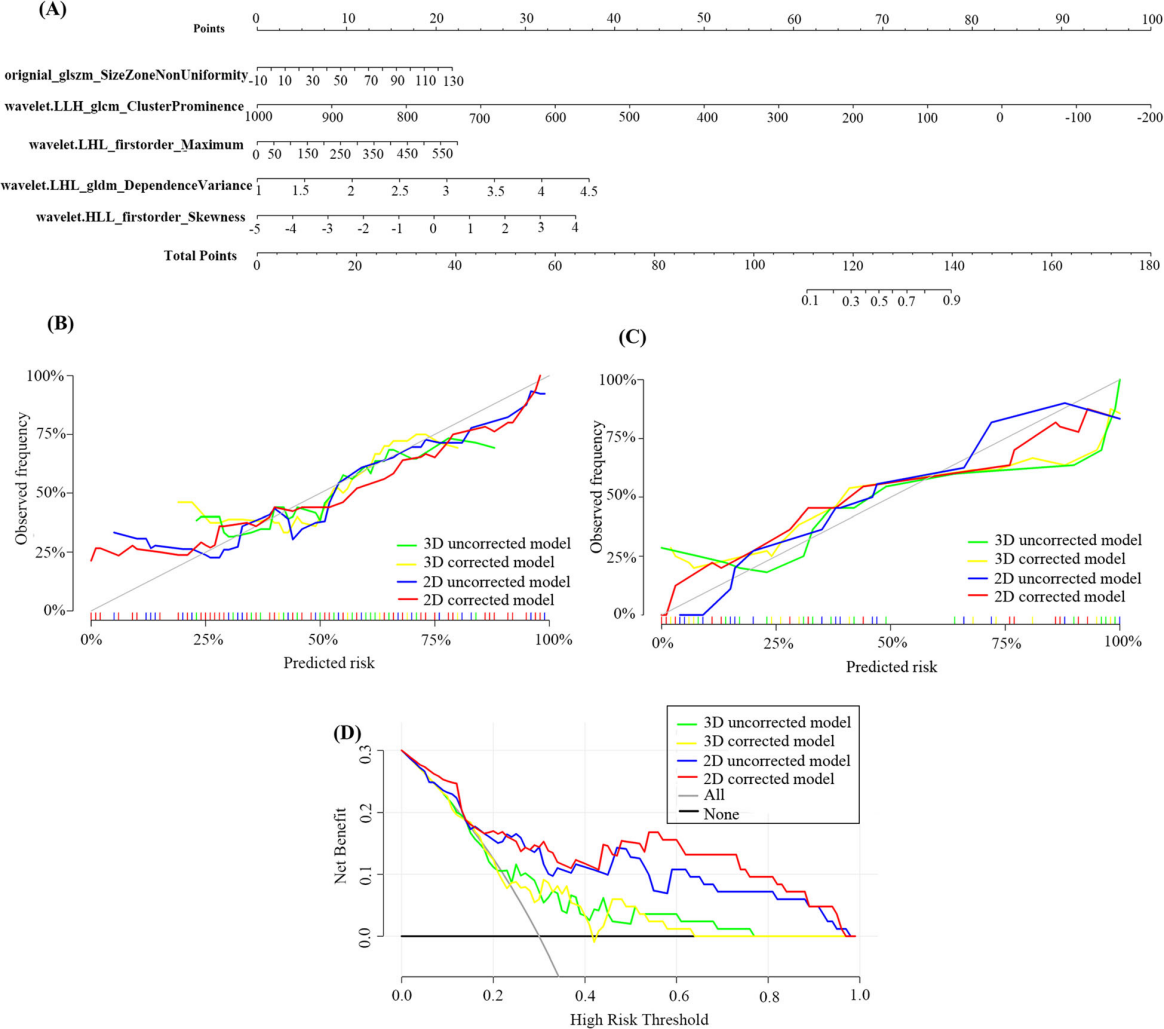
Development and performance of the radiomics nomogram. (A) Nomogram based on the 2D corrected radiomics features. (B) Calibration curves of the nomograms built on 3D uncorrected, 3D corrected, 2D uncorrected 2D corrected radiomics features in the training cohort. (C) Calibration curves of the nomograms built on 3D uncorrected, 3D corrected, 2D uncorrected 2D corrected radiomics features in the validation cohort. The calibration curves suggesting the perfect match between the actual (Y-axis) and nomogram-predicted (X-axis) responders. (D) Decision curves showed relatively good performance for the models in terms of clinical application and indicated that all the models added more benefit than either the treat-all or treat-none scheme within the threshold between 30 and 60%. Moreover, the 2D corrected model achieved the highest benefit if the threshold probability of a patient was between 50 and 70%

Quantitative nomograms for predicting the probability of responders were constructed separately for the four groups of features, of which the one based on the 2D corrected model is shown in Fig.4 (A).

The calibration curves of the four different radiomics models [Fig. 4 (B) & (C)] estimating the probability of responders demonstrated good agreement between prediction and observation in the training cohort and validation cohort. For the 2D corrected radiomics model, the Hosmer–Lemeshow test yielded a nonsignificant *P* value of 0.160 in the training cohort and 0.478 in the validation cohort, suggesting the perfect match between the actual (Y-axis) and nomogram-predicted (X-axis) responders. The 2D corrected model also achieved good discrimination performance with AUC of 0.843 (95% CI, 0.736–0.950) within the training cohort and 0.914 (95% CI, 0.775–1.000) in the validation cohort (Table 4). For 2D uncorrected model, 3D corrected model and 3D uncorrected model, AUCs were 0.794 (95% CI, 0.666–0.921), 0.658 (95% CI, 0.502–0.813) and 0.662 (95% CI, 0.509–0.816) within the training cohort and 0.898 (95% CI, 0.721–1.000), 0.670 (95% CI, 0.511–0.849) and 0.677 (95% CI, 0.499–0.850) in the validation cohort, respectively.

**Table 4:** Performance evaluation of the nomogram

	3D uncorrected model	3D corrected model	2D uncorrected model	2D corrected model
	AUC (95% CI) of the nomogram			
Training cohort	0.662	0.658	0.794	0.843
	(0.509–0.816)	(0.502–0.813)	(0.666–0.921)	(0.736–0.950)
Validation cohort	0.677	0.670	0.898	0.914
	(0.499–0.850)	(0.511–0.849)	(0.721–1.000)	(0.775–1.000)
	*P* value of Hosmer–Lemeshow test			
Training cohort	0.881	0.032	0.547	0.160
Validation cohort	0.328	0.430	0.717	0.478

Abbreviations: AUC-Area under the Receiver-Operating Characteristic Curve

### Clinical use

The decision curve was used to compare the benefit of the four different radiomics nomogram, treat-all and treat-none scheme, as shown in Fig. 4 (D). The results showed relatively good performance for the models in terms of clinical application and indicated that all the models added more benefit than either the treat-all or treat-none scheme within the threshold between 30 and 60%. Moreover, the 2D corrected model achieved the highest benefit if the threshold probability of a patient was between 50 and 70%.

## Discussion

This study aimed to evaluate the prediction efficacy of pre-therapeutic CT imaging based radiomics models in treatment response of patients with advanced ESCC receiving anti-PD-1 antibodies plus chemotherapy. In the new era of artificial intelligence (AI), radiographic characteristics automatically calculated by computer is more objective and makes more accurate quantitative analysis possible [21–24]. Our study is the first attempt to predict treatment efficacy of ICI + CT in advanced ESCC prior to treatment using CT radiomics model. The quantitative approach has the potential to identify the responders before treatment.

Due to the long-time debate on whether to use one-slice 2D annotation or whole-volume 3D annotation especially for advanced cancer [25–27], in our study, the comparison between 2D and 3D radiomic features was also performed. We found that 2D radiomic features significantly outperformed 3D features, which was similar to the reported [2^6^–^27^]. In a multicenter study of advanced gastric cancer [27], the performances of 2D and 3D CT radiomic features were compared in discriminating lymph node metastasis, lymphovascular invasion as well as pT stages’ classification. They found that 2D model outperformed 3D model with higher AUCs regarding the above three tasks despite different resampling spacings. Similar findings were also reported by another study [26] in which the prognostic prediction performances were compared between 2D and 3D CT radiomics features in patients with non-small cell lung cancer (NSCLC). They found that 2D Cox model had a higher C-index compared with 3D Cox model. The results of our study showed that 2D models performed significantly better than 3D ones, which might be attributed to more noise of 3D ROIs originated from multi-slice manual annotations and inconsistent resolutions of the transverse plane and z-plane [26–27]. Therefore, in this scenario, 2D models are recommended in ESCC radiomics researches for the better performance and time-saving annotations.

The ComBat function compensation method is a data-driven method correcting for differences in features caused by the various imaging protocols [28–29]. ComBat correction was applied in our study to control potential bias on the results caused by different CT scanning schemes such as tube voltage, reconstruction kernel, slice thickness, and in-plane resolution. This method showed efficiency in 2D models by standardizing the CT images obtained from different CT scanners, and achieved the highest AUCs in both training and validation cohort. In addition, higher net benefits could be obtained with ComBat correction in decision curve analysis, thus patients could benefit from treatment optimization and avoid unnecessary risks.

In this study, the proposed radiomics model provided potential clinical utility from the following perspectives. For patients with advanced unresectable esophageal cancer, the established radiomics model could screen out the potential responders to ICI + CT prior to treatment which would improve effeacy. On the other hand, due to the high cost of immunotherapy, preselecting the potential responders prior to treatment could reduce the economic burden to patients and maximize their benefits, which was particularly important in developing countries. In addition, as a non-invasive biomarker, CT imaging could overcome the problem of tumor heterogeneity. Some other indicators, such as PD-L1 expression, obtained by fine needle aspiration biopsy could not represent its real status in the whole tumor tissue, so the detection results might be biased due to tumor heterogeneity. Finally, in our study, we recommended 2D radiomics features because one-slice 2D annotation was a much more time-saving data processing with significantly higher prediction efficacy than that of whole-volume 3D annotation.

This study has several limitations. First, our findings deserve further extra external validation with larger sample size and inclusion of other medical centers. A large-scale study enrolling more patients is deserved and may definitely help validate and improve its applicability as an effective prediction tool for assisting treatment decision making. Second, due to the limited patient number of other histologic types of EC in our center, adenocarcinoma and signet ring cell carcinoma, therefore, were not included in the present study. This limits the application of the built model to some extent. Third, due to the limited spatial resolution of CT, there may be bias in the determination of the boundary between the lesion and the normal esophageal tissue when conducting ROIs segmentation.

## Conclusions

In conclusion, the proposed CT-based radiomics model performs well and thereby is expected to serve as an alternative tool to select the potential best responders to ICI + CT prior to treatment for patients with ESCC, thus can assist treatment decision making in the clinical setting.

## Supplementary Information

**Additional file 1.**

**Additional file 2.**

## Data Availability

The datasets supporting the conclusions of this article are included within the article.

## Abbreviations

EC: esophageal cancer
ESCC: esophageal squamous cell carcinoma
NSCLC: non-small cell lung cancer
CPS: comprehensive positive score
TPS: tumor proportion score
PD-L1: programmed death receptor ligand 1
CT: Computed tomography
AI: artificial intelligence
ICI + CT: immune-checkpoint inhibitor plus chemotherapy
BMI: Mass Index
CE-CT: Contrast enhanced computed tomography
SVM: support vector machine
DT: decision tree
LR: logistic regression
AUC: Area under the Receiver-Operating Characteristic Curves
NPV: Negative Predictive Value
PPV: Positive Predictive Value

## References

[1] Siegel RL, Miller KD, Fuchs HE, Jemal A (2021). Cancer statistics, 2021. CA Cancer J Clin.

[2] Lagergren J, Smyth E, Cunningham D, Lagergren P (2017). Oesophageal cancer. Lancet..

[3] Cowie A, Noble F, Underwood T (2014). Strategies to improve outcomes in esophageal adenocarcinoma. Expert Rev Anticancer Ther.

[4] Ajani JA, D’Amico TA, Bentrem DJ, Chao J, Corvera C, Das P, Denlinger CS, Enzinger PC, Fanta P, Farjah F, Gerdes H, Gibson M, Glasgow RE, Hayman JA, Hochwald S, Hofstetter WL, Ilson DH, Jaroszewski D, Johung KL, Keswani RN, Kleinberg LR, Leong S, Ly QP, Matkowskyj KA, McNamara M, Mulcahy MF, Paluri RK, Park H, Perry KA, Pimiento J, Poultsides GA, Roses R, Strong VE, Wiesner G, Willett CG, Wright CD, McMillian NR, Pluchino LA (2019). Esophageal and Esophagogastric junction cancers, version 2.2019, NCCN clinical practice guidelines in oncology. J Natl Compr Cancer Netw.

[5] Short MW, Burgers KG, Fry VT (2017). Esophageal Cancer. Am Fam Physician.

[6] Lian X, Zhu C, Lin H, Gao Z, Li G, Zhang N, et al.. Radiosensitization of HER2 positive esophageal cancer cells by pyrotinib. Biosci Rep. 2020 02 28;40(2). 10.1042/BSR20194167. 10.1042/BSR20194167 PMC7029153 32022229

[7] Yang YM, Hong P, Xu WW, He QY, Li B. Advances in targeted therapy for esophageal cancer. Signal Transduct Target Ther. 2020 10 07;5(1). 10.1038/s41392-020-00323-3. 10.1038/s41392-020-00323-3 PMC7542465 33028804

[8] Abdel-Rahman O, Mulder K, Easaw J. Outcomes of Ramucirumab Plus Paclitaxel Among Patients With Previously Treated Metastatic Gastric/Lower Esophageal Cancer: A Real-world Study. Am J Clin Oncol. 2021 04 01;44(4).. 10.1097/COC.0000000000000799. 10.1097/COC.0000000000000799 33625121

[9] Ferlay J, Soerjomataram I, Dikshit R, Eser S, Mathers C, Rebelo M, Parkin DM, Forman D, Bray F (2015). Cancer incidence and mortality worldwide: sources, methods and major patterns in GLOBOCAN 2012. Int J Cancer.

[10] Ott PA, Bang YJ, Piha-Paul SA, Razak ARA, Bennouna J, Soria JC (2019). T-Cell-Inflamed Gene-Expression Profile, Programmed Death Ligand 1 Expression, and Tumor Mutational Burden Predict Efficacy in Patients Treated With Pembrolizumab Across 20 Cancers: KEYNOTE-028. J Clin Oncol.

[11] Shah MA, Kojima T, Hochhauser D, Enzinger P, Raimbourg J, Hollebecque A, Lordick F, Kim SB, Tajika M, Kim HT, Lockhart AC, Arkenau HT, el-Hajbi F, Gupta M, Pfeiffer P, Liu Q, Lunceford J, Kang SP, Bhagia P, Kato K (2019). Efficacy and safety of Pembrolizumab for heavily pretreated patients with advanced, metastatic adenocarcinoma or squamous cell carcinoma of the esophagus: the phase 2 KEYNOTE-180 study. JAMA Oncol.

[12] Kojima T, Shah MA, Muro K, Francois E, Adenis A, Hsu CH, Doi T, Moriwaki T, Kim SB, Lee SH, Bennouna J, Kato K, Shen L, Enzinger P, Qin SK, Ferreira P, Chen J, Girotto G, de la Fouchardiere C, Senellart H, al-Rajabi R, Lordick F, Wang R, Suryawanshi S, Bhagia P, Kang SP, Metges JP, on behalf of the KEYNOTE-181 Investigators (2020). Randomized phase III KEYNOTE-181 study of Pembrolizumab versus chemotherapy in advanced esophageal Cancer. J Clin Oncol.

[13] Sadanand S. Immunotherapy for esophageal cancer. Nat Med. 2021 Apr 19. 10.1038/d41591-021-00022-8. 10.1038/d41591-021-00022-8 33875854

[14] Sihag S, Ku GY, Tan KS, Nussenzweig S, Wu A, Janjigian YY, Jones DR, Molena D Safety and feasibility of esophagectomy following combined immunotherapy and chemoradiotherapy for esophageal cancer. J Thorac Cardiovasc Surg 2021 03;161(3). 10.1016/j.jtcvs.2020.11.106, 3, 843.e1. 10.1016/j.jtcvs.2020.11.106 PMC7889638 33485662

[15] Bando H, Kotani D, Tsushima T, Hara H, Kadowaki S, Kato Ke, Chin K, Yamaguchi K, Kageyama SI, Hojo H, Nakamura M, Tachibana H, Wakabayashi M, Fukutani M, Togashi Y, Fuse N, Nishikawa H, Kojima T TENERGY: multicenter phase II study of Atezolizumab monotherapy following definitive Chemoradiotherapy with 5-FU plus cisplatin in patients with unresectable locally advanced esophageal squamous cell carcinoma. BMC Cancer 2020;20(1):336. 10.1186/s12885-020-06716-5. 10.1186/s12885-020-06716-5 PMC7168951 32312286

[16] Fuchs CS, Doi T, Jang RW, Muro K, Satoh T, Machado M, et al. Safety and Efficacy of Pembrolizumab Monotherapy in Patients with Previously Treated Advanced Gastric and Gastroesophageal Junction Cancer: Phase 2 Clinical KEYNOTE-059 Trial. 2018;4(5):e180013. 10.1001/jamaoncol.2018.0013. 10.1001/jamaoncol.2018.0013 PMC5885175 29543932

[17] Trebeschi S, Drago SG, Birkbak NJ, Kurilova I, Calin AM, Pizzi AD, et al. Predicting response to cancer immunotherapy using noninvasive radiomic biomarkers.. Ann Oncol. 2019;30(6):998–1004. 10.1093/annonc/mdz108. 10.1093/annonc/mdz108 PMC6594459 30895304

[18] Sun R, Sundahl N, Hecht M, Putz F, Lancia A, Rouyar A, et al. Radiomics to predict outcomes and abscopal response of patients with cancer treated with immunotherapy combined with radiotherapy using a validated signature of CD8 cells. J Immunother Cancer. 2020;8(2):e001429. 10.1136/jitc-2020-001429. 10.1136/jitc-2020-001429 PMC7668366 33188037

[19] Du Y, Qi Y, Jin Z, Tian J, et al. Noninvasive imaging in cancer immunotherapy: The way to precision medicine. Cancer Lett. 2019; 466:13–22. 10.1016/ j.canlet.2019.08.009. 10.1016/j.canlet.2019.08.009 31499119

[20] Eisenhauer EA, Therasse P, Bogaerts J, Schwartz LH, Sargent D, Ford R, et al. New response evaluation criteria in solid tumours: revised RECIST guideline (version 1.1). Eur J Cancer. 2009; 45(2):228–47. 10.1016/j.ejca.2008.10.026. 10.1016/j.ejca.2008.10.026 19097774

[21] Hosny A, Parmar C, Quackenbush J, Schwartz LH, Aerts HJW, et al.Artificial intelligence in radiology. Nat Rev Cancer. 2018;18(8):500–10.10.1038/s41568-018-0016-5. 10.1038/s41568-018-0016-5 PMC6268174 29777175

[22] Aerts HJ. The Potential of Radiomic-Based Phenotyping in Precision Medicine: A Review. JAMA Oncol. 2016;2(12):1636–42. 10.1001/jamaoncol.2016.2631. 10.1001/jamaoncol.2016.2631 27541161

[23] Ji Z, Cui Y, Peng Z, Gong JF, Zhu HT, Zhang XT. Use of Radiomics to Predict Response to Immunotherapy of Malignant Tumors of the Digestive System. Med Sci Monit. 2020;26:e924671. 10.12659/MSM.924671. 10.12659/MSM.924671 PMC7586759 33077705

[24] Park KJ, Lee JL, Yoon SK, Heo CH, Park BW, Kim JK,et al. Radiomics-based prediction model for outcomes of PD-1/PD-L1 immunotherapy in metastatic urothelial carcinoma. Eur Radiol. 2020;30(10):5392–403. 10.1007/s00330-020-06847-0. 10.1007/s00330-020-06847-0 32394281

[25] Yang L, Yang J, Zhou X, Huang L, Zhao W, Wang T,et al. Development of a radiomics nomogram based on the 2D and 3D CT features to predict the survival of non-small cell lung cancer patients. 2019;29(5):2196–206. 10.1007/s00330-018-5770-y. 10.1007/s00330-018-5770-y 30523451

[26] Shen C, Liu Z, Guan M, Song J, Lian Y, Wang S, et al. 2D and 3D CT Radiomics Features Prognostic Performance Comparison in Non-Small Cell Lung.Transl Oncol.2017;10(6):886–94. 10.1016/j.tranon.2017.08.007. 10.1016/j.tranon.2017.08.007 PMC5605492 28930698

[27] Meng L, Dong D, Chen X, Fang M, Wang R, Li J, et al. 2D and 3D CT Radiomic Features Performance Comparison in Characterization of Gastric Cancer: A Multi-Center Study. IEEE J Biomed Health Inform. 2021;25(3):755-63. 10.1109/JBHI.2020.3002805. 10.1109/JBHI.2020.3002805 32750940

[28] Orlhac F, Frouin F, Nioche C, Ayache N, Buvat I. Validation of A Method to Compensate Multicenter Effects Affecting CT Radiomics. Radiology. 2019; 291(1):53–9. 10.1148/radiol.2019182023 10.1148/radiol.2019182023 30694160

[29] Ligero M, Jordi-Ollero O, Bernatowicz K, Garcia-Ruiz A, Delgado-Munoz E, Levia D, et al. Minimizing acquisition-related radiomics variability by image resampling and batch effect correction to allow for large- scale data analysis. Eur Radiol. 2021;31(3):1460–70. 10.1007/s00330-020-07174-0. 10.1007/s00330-020-07174-0 PMC7880962 32909055

