# Supplementary material for: Predicting response to immunotherapy plus chemotherapy in patients with esophageal squamous cell carcinoma using non-invasive Radiomic biomarkers
Source: BMC Cancer. 2021 Oct 30;21:1167. doi: 10.1186/s12885-021-08899-x (PMC8557514; doi:10.1186/s12885-021-08899-x)
Supplement: Supplementary file 1 — Additional file 1. [file 12885_2021_8899_MOESM1_ESM.docx]

| Supplementary Table 1. Detailed information of the CT scanners. | | | |
| --- | --- | --- | --- |
| **Manufacture** | **Country** | **Slice thickness and spacing** | **kVp** |
| GE MEDICAL SYSTEMS | America | 1.25 | 120 |
| Philips | The Netherlands | 1 | 120 |
| SIEMENS | Germany | 1 | 90/100 |
| TOSHIBA | Japan | 2 | 120 |
| UNITED IMAGING HEALTHCARE | China | 1 | 120 |
