# Supplementary material for: Predicting response to immunotherapy plus chemotherapy in patients with esophageal squamous cell carcinoma using non-invasive Radiomic biomarkers
Source: BMC Cancer. 2021 Oct 30;21:1167. doi: 10.1186/s12885-021-08899-x (PMC8557514; doi:10.1186/s12885-021-08899-x)
Supplement: Supplementary file 2 — Additional file 2. [file 12885_2021_8899_MOESM2_ESM.docx]

| Supplementary Table 2. AUCs (95%CI) of the different algorithms among the four models in the training cohorts. | | | | | |
| --- | --- | --- | --- | --- | --- |
| **Models** | **SVM** | **KN** | **RF** | **DT** | **LR** |
| 3D uncorrected | 0.620 | 0.602 | 0.701 | 0.683 | 0.644 |
|  | (0.601-0.633) | (0.590-0.622) | (0.694-0.723) | (0.675-0.697) | (0.640-0.668) |
| 3D corrected | 0.601 | 0.590 | 0.661 | 0.646 | 0.640 |
|  | (0.582-0.620) | (0.581-0.606) | (0.642-0.687) | (0.637-0.655) | (0.620-0.662) |
| 2D uncorrected | 0.780 | 0.702 | 0.719 | 0.680 | 0.762 |
|  | (0.763-0.806) | (0.690-0.721) | (0.710-0.737) | (0.676-0.704) | (0.761-0.780) |
| 2D corrected | 0.804 | 0.731 | 0.750 | 0.705 | 0.783 |
|  | (0.800-0.822) | (0.722-0.743) | (0.741-0.780) | (0.690-0.716) | (0.784-0.807) |
| Abbreviations: AUCs-Area under the Receiver-Operating Characteristic Curves, 95%CI-95% Confidence Interval, SVM-Support Vector Machine, KN-K Nearest Neighbors, RF-Random Forest, DT-Decision Tree, LR-Logistic Regression. | | | | | |
